# Supplementary material for: Sex expression and floral diversity in Jatropha curcas: a population study in its center of origin
Source: PeerJ. 2016 May 24;4:e2071. doi: 10.7717/peerj.2071 (PMC4888319; doi:10.7717/peerj.2071)
Supplement: Table S1 — Accessions of Jatropha curcas L. from the Jatropha Germplasm Bank of the Universidad Autónoma de Chiapas, grouped in populations according its geographical origin. [file peerj-04-2071-s001.docx]

**Table S1**. Accessions of *Jatropha curcas* L. from the Jatropha Germplasm Bank of the Universidad Autónoma de Chiapas, studied in its floral traits.

| **Municipality (Accession** | **Number of accessions** | **Data from the original collection site** | | |
| --- | --- | --- | --- | --- |
|  |  | **Latitude (North)** | **Longitude (West)** | **Population** |
| Acapetahua (ACA) | 2 | 15°10.300' | 92°35.100' | Soconusco |
| Cacahoatán (CAC) | 5 | 14°59.022' | 92°10.051' | Soconusco |
| Huixtla (HUX) | 8 | 15°05.115' | 92°29.146' | Soconusco |
| Mapastepec (MAP) | 7 | 15°25.505' | 92°53.854' | Soconusco |
| Puerto Chiapas (PC) | 11 | 14°43.742' | 92°25.935' | Soconusco |
| Suchiate (SCH) | 4 | 14°40.057' | 92°10.071' | Soconusco |
| Tapachula (TAP) | 1 | 14°54.290' | 92°15.380' | Soconusco |
| Arriaga (ARR) | 11 | 16°11.231' | 93°64.816' | Istmo |
| Pijijiapan (PIJ) | 4 | 15°55.561' | 92°59.842' | Istmo |
| Tonalá (TON) | 2 | 16°03.430' | 93°50.782' | Istmo |
| La Concordia (CCR) | 4 | 16°06.663' | 92°41.035' | Frontera |
| Cd. Cuauhtémoc (CDCU) | 5 | 15°40.473' | 92°00.129' | Frontera |
| Chicomuselo (CHIC) | 1 | 15°44.623' | 92°16.722' | Frontera |
| Comalapa (COM) | 5 | 15°39.030' | 92°08.170' | Frontera |
| Lagos de Colón (LACO) | 1 | 15°50.335' | 91°54.238' | Frontera |
| Rizo de Oro (RIZ) | 3 | 17° 57.981' | 92°28.824' | Frontera |
| Berriozábal (BERR) | 1 | 16°47.562' | 93°16.191' | Centro |
| Ixtapa (IXT) | 1 | 16°47.220' | 92°54.618' | Centro |
| Jiquipilas (JIQ) | 2 | 16°40.012' | 93°39.242' | Centro |
| Ocozocuatla (OCZ) | 1 | 16°46.243' | 93°23.641' | Centro |
| Pujiltic (PUJ) | 6 | 16°16.430' | 92°17,850' | Frailesca |
| Villa Corzo (VCO) | 3 | 16°10,171' | 93°16,059' | Frailesca |
| Villa Flores (VIF) | 2 | 16°19,475' | 93°20,976' | Frailesca |
| Villa de las Rosas (VLR) | 1 | 16°19.243' | 92°20,578' | Frailesca |
| El coco (ECO) | 2 | 15°43.366' | 96°33.971' | Oaxaca |
| Piedra Ancha (PA) | 1 | 16°11.238' | 97°44.825' | Oaxaca |
| Pinotepa Nacional (PIN) | 1 | 16°19.306' | 97°53.564' | Oaxaca |
| Zimatán (ZIM) | 1 | 15°50.564' | 96°00.154' | Oaxaca |
| Apatzingán (APA) | 1 | 19°04.247' | 102°22.098' | Michoacán |
| Copala (COP) | 2 | 16°37.433' | 99°00.749' | Guerrero |
| Pueblillo (PUEB) | 1 | 20º15.110' | 97º15.300' | Veracruz |
| Yautepec (YAU) | 1 | 18°53.000' | 99°04.000' | Morelos |
| Guatemala (GUA) | 3 | 14°36.275' | 90°29.083' | Guatemala |
