# Supplementary material for: Sex expression and floral diversity in Jatropha curcas: a population study in its center of origin
Source: PeerJ. 2016 May 24;4:e2071. doi: 10.7717/peerj.2071 (PMC4888319; doi:10.7717/peerj.2071)
Supplement: Table S3 — Contributions of floral traits to principal components as generated in a discriminant analysis of 103 Jatropha curcas L. accessions from Southern Mexico. [file peerj-04-2071-s002.docx]

**Table S3**. Contributions of floral traits to principal components as generated in a discriminant analysis of 103 *Jatropha curcas* L. accessions from Southern Mexico.

| **Correlations Variables / Components** | | | | | | | |
| --- | --- | --- | --- | --- | --- | --- | --- |
|  | **C1A** | **C2A** | **C3A** | **C4A** | **C5A** | **C6A** | **C7A** |
| Male flower diameter | 0.470 | -0.137 | -0.118 | 0.197 | -0.025 | -0.029 | 0.197 |
| Male sepal lenght | 0.287 | -0.202 | -0.085 | -0.075 | 0.131 | -0.197 | 0.208 |
| Male sepal width | -0.061 | -0.084 | 0.217 | 0.362 | -0.152 | -0.337 | 0.246 |
| Male petal lenght | 0.121 | -0.121 | -0.131 | 0.184 | 0.021 | -0.429 | 0.383 |
| Male petal width | 0.301 | -0.122 | 0.049 | 0.016 | 0.069 | -0.445 | 0.166 |
| Male nectary length | 0.311 | 0.393 | -0.042 | 0.053 | 0.141 | -0.293 | 0.572 |
| Male nectary thickness | 0.147 | -0.097 | -0.003 | 0.024 | 0.134 | -0.224 | 0.534 |
| Filament lenght | 0.053 | 0.267 | -0.077 | 0.063 | -0.302 | -0.053 | 0.490 |
| Filament thickness | 0.065 | -0.039 | -0.247 | 0.152 | 0.185 | -0.156 | 0.454 |
| Anther lenght | 0.275 | -0.054 | -0.034 | 0.172 | 0.099 | -0.431 | 0.087 |
| Anther thickness | -0.106 | 0.253 | 0.007 | -0.244 | 0.061 | -0.219 | -0.026 |
| Pollen diameter | 0.108 | -0.005 | -0.071 | -0.041 | 0.056 | -0.023 | 0.366 |
| Trichomes in male flowers | -0.055 | 0.313 | -0.112 | 0.439 | 0.174 | -0.051 | 0.146 |
| Female/hermaphrodite flower diameter | 0.396 | -0.028 | 0.021 | 0.063 | 0.069 | 0.132 | 0.058 |
| Female/hermaphrodite sepal length | 0.407 | 0.232 | -0.028 | -0.003 | 0.002 | 0.296 | -0.062 |
| Female/hermaphrodite sepal width | 0.329 | -0.029 | -0.182 | 0.196 | 0.079 | 0.132 | -0.299 |
| Female/hermaphrodite petal length | 0.397 | -0.015 | -0.019 | 0.087 | -0.001 | 0.098 | 0.095 |
| Female/hermaphrodite petale width | 0.349 | -0.043 | -0.071 | 0.109 | -0.136 | -0.053 | -0.191 |
| Female/hermaphrodite nectary length | 0.209 | 0.363 | 0.119 | 0.142 | 0.012 | 0.064 | -0.066 |
| Female/hermaphrodite nectary thickness | 0.282 | 0.290 | 0.107 | 0.135 | -0.260 | 0.048 | -0.047 |
| Pistil lenght | 0.231 | 0.300 | 0.009 | -0.098 | 0.108 | 0.122 | 0.003 |
| Pistil thickness | 0.360 | 0.321 | 0.188 | 0.002 | 0.127 | -0.046 | -0.031 |
| Ovary lenght | 0.162 | 0.420 | 0.246 | -0.028 | -0.134 | 0.017 | -0.142 |
| Ovary thickness | 0.225 | 0.238 | 0.233 | 0.127 | 0.062 | 0.170 | -0.070 |
| Ovule lenght | 0.047 | -0.056 | -0.333 | 0.017 | -0.294 | -0.191 | -0.100 |
| Ovule thickness | 0.090 | 0.092 | -0.104 | -0.120 | -0.226 | -0.085 | -0.011 |
| Trichomes in female/hermaphrodite flowers | 0.175 | 0.006 | 0.339 | 0.321 | 0.057 | 0.138 | 0.042 |
