# Supplementary material for: Sex expression and floral diversity in Jatropha curcas: a population study in its center of origin
Source: PeerJ. 2016 May 24;4:e2071. doi: 10.7717/peerj.2071 (PMC4888319; doi:10.7717/peerj.2071)
Supplement: Table S4 — Contributions of floral traits to principal components as generated in a discriminant analysis of Jatropha curcas L. accessions from Chiapas, Mexico. [file peerj-04-2071-s003.docx]

**Table S4**. Contributions of floral traits to principal components as generated in a discriminant analysis of *Jatropha curcas* L. accessions from Chiapas, Mexico.

| **Correlations Variables / Components** | | | | |
| --- | --- | --- | --- | --- |
|  | **C1B** | **C2B** | **C3B** | **C4B** |
| Male flower diameter | 0.442 | -0.018 | -0.217 | 0.190 |
| Male sepal lenght | 0.370 | 0.021 | -0.013 | 0.261 |
| Male sepal width | -0.031 | -0.225 | -0.168 | 0.359 |
| Male petal lenght | 0.192 | 0.078 | -0.220 | 0.522 |
| Male petal width | 0.362 | 0.022 | 0.027 | 0.301 |
| Male nectary length | 0.184 | 0.305 | 0.064 | 0.671 |
| Male nectary thickness | 0.195 | -0.029 | -0.028 | 0.563 |
| Filament lenght | -0.123 | 0.174 | -0.004 | 0.498 |
| Filament thickness | 0.109 | 0.125 | -0.259 | 0.485 |
| Anther lenght | 0.323 | 0.083 | -0.129 | 0.238 |
| Anther thickness | -0.125 | 0.252 | 0.253 | 0.063 |
| Pollen diameter | 0.099 | 0.041 | -0.002 | 0.348 |
| Trichomes in male flowers | -0.138 | 0.159 | -0.359 | 0.194 |
| Female/hermaphrodite flower diameter | 0.361 | -0.065 | -0.024 | -0.004 |
| Female/hermaphrodite sepal length | 0.246 | 0.111 | 0.068 | -0.183 |
| Female/hermaphrodite sepal width | 0.312 | 0.081 | -0.263 | -0.346 |
| Female/hermaphrodite petal length | 0.348 | -0.026 | -0.059 | 0.048 |
| Female/hermaphrodite petale width | 0.307 | 0.055 | -0.102 | -0.175 |
| Female/hermaphrodite nectary length | 0.046 | 0.100 | 0.064 | -0.090 |
| Female/hermaphrodite nectary thickness | 0.086 | 0.075 | 0.069 | -0.077 |
| Pistil lenght | 0.111 | 0.186 | 0.178 | -0.042 |
| Pistil thickness | 0.255 | 0.100 | 0.233 | -0.019 |
| Ovary lenght | -0.039 | 0.098 | 0.312 | -0.159 |
| Ovary thickness | 0.101 | -0.093 | 0.105 | -0.144 |
| Ovule lenght | 0.032 | 0.283 | -0.199 | -0.017 |
| Ovule thickness | 0.015 | 0.193 | 0.085 | 0.020 |
| Trichomes in female/hermaphrodite flowers | 0.218 | -0.259 | 0.008 | 0.008 |
