# Supplementary material for: Sex expression and floral diversity in Jatropha curcas: a population study in its center of origin
Source: PeerJ. 2016 May 24;4:e2071. doi: 10.7717/peerj.2071 (PMC4888319; doi:10.7717/peerj.2071)
Supplement: Table S2 — Coding floral characters of Jatropha curcas L. accessions from the Jatropha Germplasm Bank of the Universidad Autónoma de Chiapas. [file peerj-04-2071-s004.docx]

**Table S2.** Coding floral characters of *Jatropha curcas* L. accessions from the Jatropha Germplasm Bank of the Universidad Autónoma de Chiapas.

| **Diameter of male flower** | | **Length of male sepal** | | **Width of male sepal** | |
| --- | --- | --- | --- | --- | --- |
| Actual value (µm) | Encoded value | Actual value (µm) | Encoded value | Actual value (µm) | Encoded value |
| 0 | 0 | 0 | 0 | 0 | 0 |
| <6000 | 1 | <3000 | 1 | <2000 | 1 |
| <7000 | 2 | <4000 | 2 | <3000 | 2 |
| <8000 | 3 | <5000 | 3 | <4000 | 3 |
| <9000 | 4 | <6000 | 4 | <5000 | 4 |
| <10000 | 5 | <7000 | 5 | <6000 | 5 |
| <11000 | 6 |  |  |  |  |
| **Length of male petal** | | **Width of male petal** | | **Length of male nectary** | |
| Actual value (µm) | Encoded value | Actual value (µm) | Encoded value | Actual value (µm) | Encoded value |
| 0 | 0 | 0 | 0 | 0 | 0 |
| ˂5000 | 1 | <2000 | 1 | <1000 | 1 |
| ˂6000 | 2 | <3000 | 2 | <2000 | 2 |
| ˂7000 | 3 | < 4000 | 3 |  |  |
| ˂8000 | 4 | < 5000 | 4 |  |  |
|  |  | < 6000 | 5 |  |  |
| **Width of male nectary** | | **Length of filament** | | **Thickness of filament** | |
| Actual value (µm) | Encoded value | Actual value (µm) | Encoded value | Actual value (µm) | Encoded value |
| 0 | 0 | 0 | 0 | 0 | 0 |
| <1000 | 1 | <3000 | 1 | <200 | 1 |
| <2000 | 2 | <4000 | 2 | <300 | 2 |
|  |  | <5000 | 3 | <400 | 3 |
|  |  | <6000 | 4 |  |  |
|  |  | <7000 | 5 |  |  |
|  |  | <8000 | 6 |  |  |
| **Length of anther** | | **Width of anther** | | **Diameter of pollen** | |
| Actual value (µm) | Encoded value | Actual value (µm) | Encoded value | Actual value (µm) | Encoded value |
| 0 | 0 | 0 | 0 | 0 | 0 |
| < 1000 | 1 | < 800 | 1 | 10-30 | 1 |
| < 2000 | 2 | < 1000 | 2 | 30-40 | 2 |
|  |  | \|  \| 3 \| \| --- \| --- \| |  | >40 | 3 |
| **Diameter of female flower** | | **Length of female sepal** | | **Width of female sepal** | |
| Actual value (µm) | Encoded value | Actual value (µm) | Encoded value | Actual value (µm) | Encoded value |
| 0 | 0 | 0 | 0 | 0 | 0 |
| <7000 | 1 | <5000 | 1 | <3000 | 1 |
| <9000 | 2 | <7000 | 2 | <4000 | 2 |
| <11000 | 3 | <9000 | 3 | <5000 | 3 |
| <13000 | 4 | <11000 | 4 | <6000 | 4 |
| <15000 | 5 |  |  | <7000 | 5 |
| **Length of female petal** | | **Width of female Petal** | | **Length of female nectary** | |
| Actual value (µm) | Encoded value | Actual value (µm) | Encoded value | Actual value (µm) | Encoded value |
| 0 | 0 | 0 | 0 | 0 | 0 |
| <6000 | 1 | <3000 | 1 | <1000 | 1 |
| <7000 | 2 | <4000 | 2 | <2000 | 2 |
| <8000 | 3 |  |  |  |  |
| <9000 | 4 |  |  |  |  |
| <10000 | 5 |  |  |  |  |
| **Width of female nectary** | | **Length of pistil** | | **Thickness of pistil** | |
| Actual value (µm) | Encoded value | Actual value (µm) | Encoded value | Actual value (µm) | Encoded value |
| 0 | 0 | 0 | 0 | 0 | 0 |
| <1000 | 1 | <2000 | 1 | <500 | 1 |
| <2000 | 2 | <3000 | 2 | <1000 | 2 |
|  |  | < 4000 | 3 |  |  |
| **Length of ovary** | | **Width of ovary** | | **Length of ovule** | |
| Actual value (µm) | Encoded value | Actual value (µm) | Encoded value | Actual value (µm) | Encoded value |
| 0 | 0 | 0 | 0 | 0 | 0 |
| <2000 | 1 | <2000 | 1 | <400 | 1 |
| <3000 | 2 | <3000 | 2 | <500 | 2 |
| < 4000 | 3 | < 4000 | 3 | <600 | 3 |
|  |  |  |  | <700 | 4 |
|  |  |  |  | <800 | 5 |
| **Width of ovule** | |  | |  | |
| Actual value (µm) | Encoded value |  |  |  |  |
| 0 | 0 |  |  |  |  |
| <400 | 1 |  |  |  |  |
| <500 | 2 |  |  |  |  |
| <600 | 3 |  |  |  |  |
