# Supplementary figures and images for: Sex expression and floral diversity in Jatropha curcas: a population study in its center of origin
Source: PeerJ. 2016 May 24;4:e2071. doi: 10.7717/peerj.2071 (PMC4888319; doi:10.7717/peerj.2071)

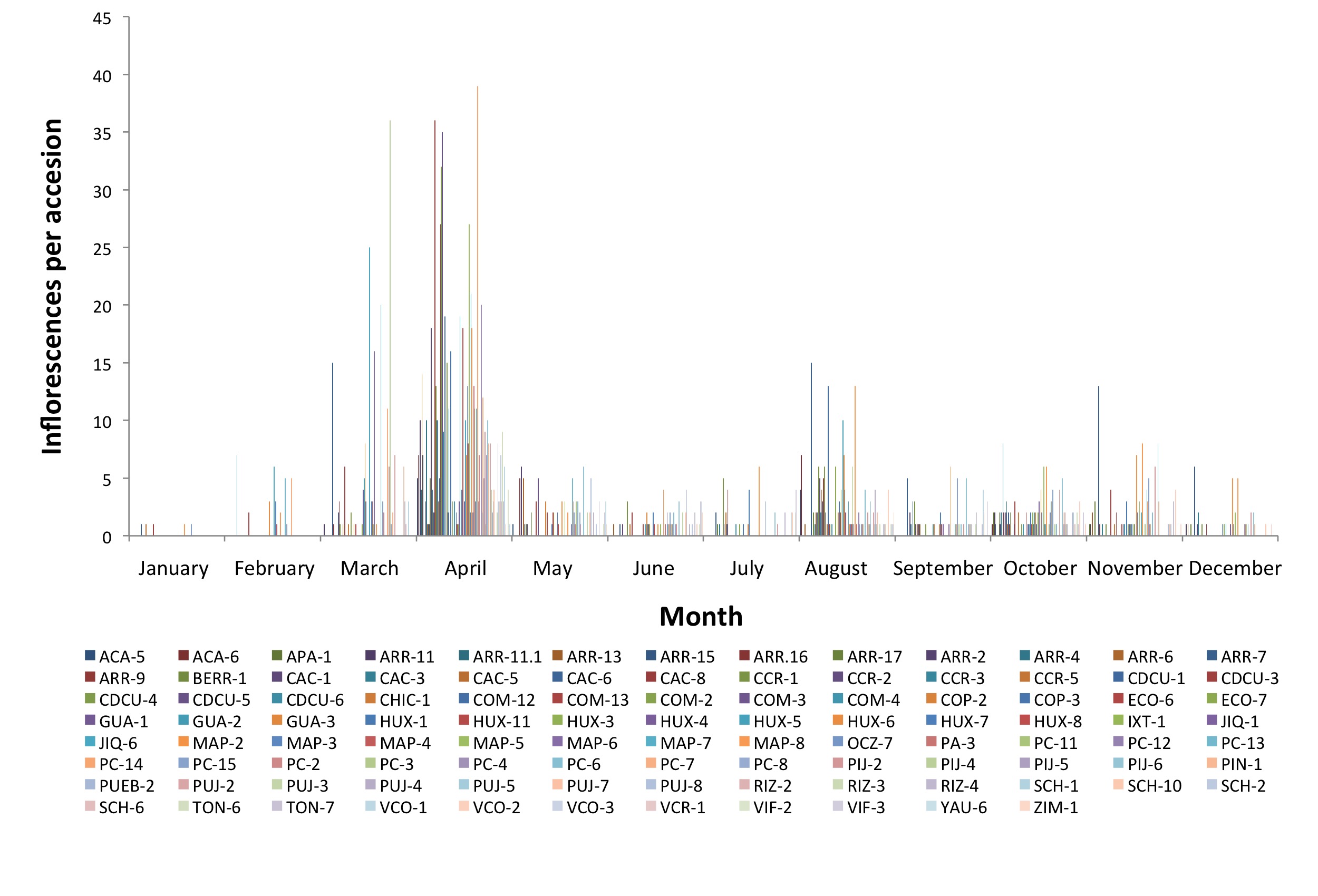

Supplement: Figure S1 — Flowering dynamics of 103 accessions of Jatropha curcas L. from the Jatropha Germplasm Bank of the Autonomous University of Chiapas, Mexico. [file peerj-04-2071-s005.jpg]

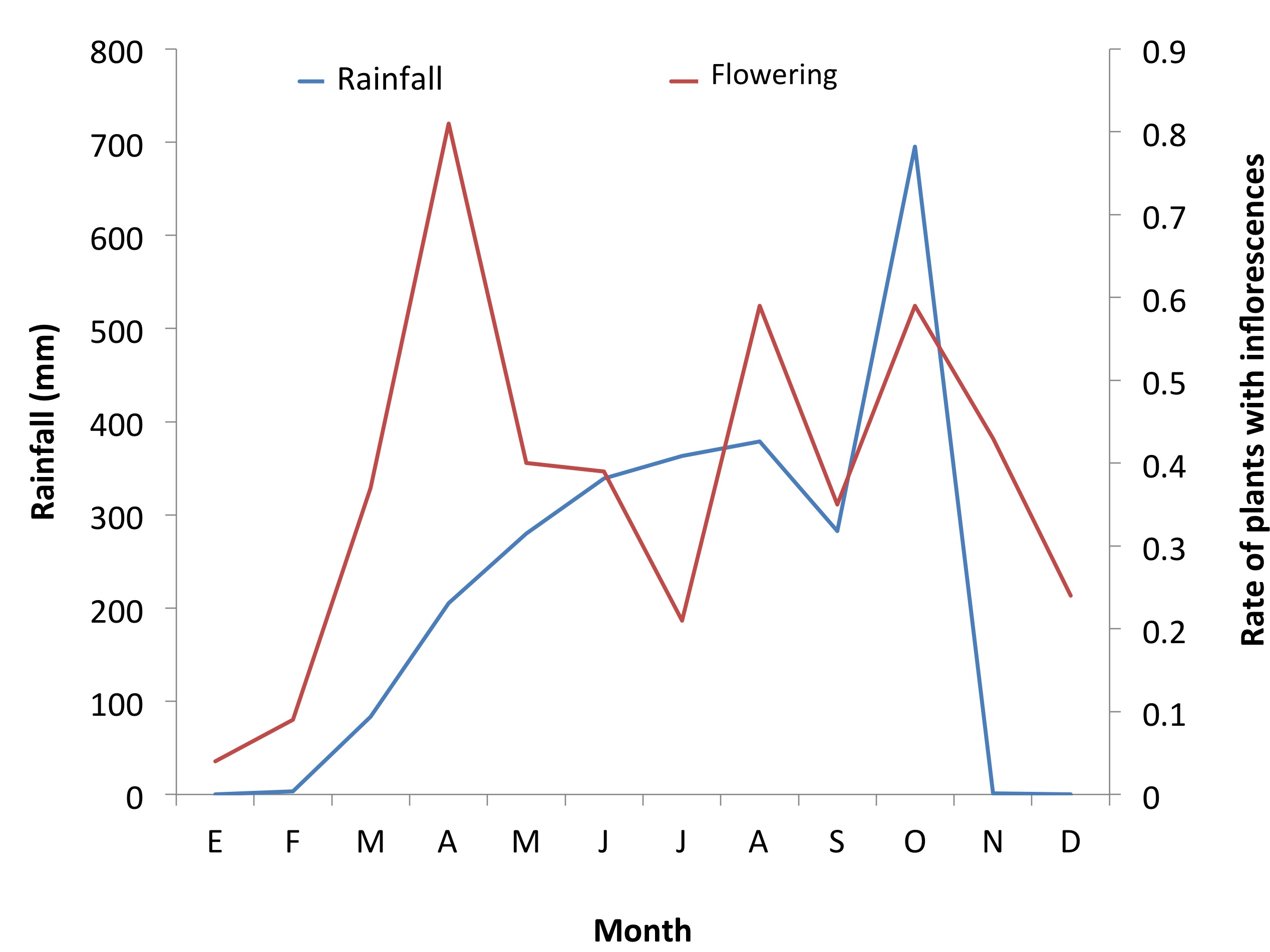

Supplement: Figure S2 — Relationship between the proportion of flowering accessions of Jatropha curcas L. from the Jatropha Germplasm Bank of the Autonomous University of Chiapas and rainfall in Tapachula, Chiapas, México. [file peerj-04-2071-s006.jpg]
